# Selective Expansion of TCR Vβ2^+^ Tregs and Impaired Lymphocyte TSST‐1 Responsiveness in Bullous Pemphigoid Patients Colonised by *Staphylococcus aureus*

**DOI:** 10.1111/exd.70355

**Published:** 2026-09-21

**Authors:** Tian Y. Chen, Tyler P. Crowe, Maryam Fakhimi, Mia Poleksíc, Samuel H. Kilgore, Patrick M. Schlievert, Janet A. Fairley, Kelly N. Messingham

**Affiliations:** ^1^ Department of Dermatology, Carver College of Medicine University of Iowa Iowa USA; ^2^ Hennepin County Medical Center Minneapolis Minnesota USA; ^3^ Mayo Clinic College of Medicine and Science Rochester Minnesota USA; ^4^ Department of Microbiology and Immunology, Carver College of Medicine University of Iowa Iowa USA

**Keywords:** pemphigoid bullous, *Staphylococcus aureus*, superantigen, T‐lymphocytes, TSST‐1

## Abstract

Bullous pemphigoid (BP) is an autoimmune blistering disease of the elderly who are frequently colonised by *Staphylococcus aureus* , particularly strains producing the superantigen toxic shock syndrome toxin‐1 (TSST‐1). Given the ability of TSST‐1 to engage the T cell receptor (TCR) Vβ2 chain, we investigated whether superantigen (SAg) exposure in BP is associated with alterations in peripheral T cell populations. CD4^+^ T cells were isolated from peripheral blood mononuclear cells (PBMCs) from BP patients and age‐matched healthy controls. T cell responsiveness to TSST‐1, staphylococcus enterotoxin G (SEG), and staphylococcus enterotoxin‐like‐I (SEl‐I) was assessed via proliferation assays, and the TCR Vβ repertoire was analysed by RT‐qPCR of bulk CD4^+^ T cells and flow cytometry of conventional CD4^+^, CD8^+^, and regulatory T cell (Treg, CD4^+^FoxP3^+^) subsets. Contrary to expectations, proliferative responses to TSST‐1 were significantly lower in BP samples than in controls, while responses to SEG and SEl‐I were comparable. RT‐qPCR revealed no expansion of Vβ2^+^ in bulk CD4^+^ T cells, and surface phenotyping detected no enrichment of Vβ2^+^ cells within conventional CD4^+^ or CD8^+^ T populations. In contrast, Vβ2^+^ cells were selectively enriched within the Treg compartment. These findings indicate that peripheral T cell responses to TSST‐1 are attenuated in BP and are associated with subset‐specific skewing of the TCR repertoire. These findings are consistent with increased regulatory features in the peripheral immune compartment that may limit systemic SAg reactivity. Studies examining lesional skin immune populations are needed to determine whether local T cell responses differ from those observed in peripheral blood.

## 1 Introduction

Bullous pemphigoid (BP) is an autoimmune blistering disease that primarily affects the elderly and is characterised by pruritic, inflamed skin and the development of large subepidermal blisters [1]. BP significantly impairs quality of life and is associated with increased mortality [2]. BP pathogenesis is mediated by the downstream events following autoantibody binding to BP180 (collagen XVII) in the skin [3]. However, like other autoimmune diseases, emerging evidence suggests that immune activation triggered by environmental pathogens may contribute to the disease course [4].

In the skin, enrichment of *Staphylococcus aureus* ( *S. aureus* ) is associated with several inflammatory and autoimmune diseases, including multiple sclerosis, Crohn's disease, systemic lupus erythematosus, and atopic dermatitis [5, 6, 7, 8, 9, 10]. The deleterious effects of *S. aureus* are mediated by secreted superantigen (SAg) toxins, which directly crosslink the T cell receptor (TCR) Vβ chain with major histocompatibility complex (MHC) Class II molecules, leading to polyclonal activation of T cells and antigen‐presenting cells (APCs), resulting in immune dysregulation, including cytokine storm, T cell anergy or apoptosis [11, 12, 13], and autoreactive T and B cell responses [14]. Toxic shock syndrome toxin‐1 (TSST‐1) is the best‐known SAg, as it is the primary cause of death in toxic shock syndrome and is linked to the severity of several autoimmune and inflammatory diseases [15, 16].

Recently, BP patients have been characterised by bacterial dysbiosis featuring enrichment of *S. aureus* [17, 18]. A 2022 study by our group found that 85.7% of BP lesions were colonised by *S. aureus* , significantly exceeding colonisation rates observed on the skin or in the nares of healthy controls (10.7% each), where coagulase‐negative staphylococci predominated [17]. Moreover, 92% of *S. aureus* isolates from BP lesions expressed TSST‐1, compared to < 10% in healthy individuals. Complementary high‐resolution profiling studies revealed a shift from protective to pathogenic staphylococcal species at the margins of BP lesions [18]. The present study investigates whether *S. aureus* colonisation in BP patients is associated with SAg‐mediated alterations in the peripheral T cell compartment.

## 2 Materials and Methods

### 2.1 Patient Population

This study was approved by the University of Iowa Institutional Review Board (IRB# 2016077048, 202 008 473) and conducted in accordance with the Declaration of Helsinki. Patients with suspected or confirmed BP evaluated at the University of Iowa Health Care (UIHC) between March 2017 and June 2025 were offered enrollment, and written informed consent was obtained. As UIHC is a tertiary referral centre, patients may have been seen by outside dermatologists before being referred to UIHC's Blistering Disease clinic.

Inclusion criteria were clinical features consistent with BP (cutaneous blistering, erythematous/urticarial plaques), histologic evidence of sub‐epidermal blistering, and either linear deposition of IgG and/or C3 at the basement membrane zone of biopsied skin or circulating BP180‐specific IgG.

Healthy controls, age‐matched to the BP cohort, were recruited from patients attending routine clinic visits, primarily for skin checks and evaluation of benign lesions. Controls had no history of autoimmune disease and no reported use of antibiotics or immunosuppressive medications in the two weeks preceding enrollment.

Bacterial swabs and peripheral blood were collected from all patients, and demographic information, laboratory results, and medications taken in the two weeks preceding enrollment were recorded (Table 1). Participants were assigned numerical codes at enrollment, and investigators performing laboratory assays were blinded to clinical group assignment and patient information.

**TABLE 1:** Clinical characteristics of BP patients and healthy controls and assays performed using PBMC.

Bullous pemphigoid patients									Healthy Controls			
Patient ID	Assays^b^	Sex^c^	Age, years	Lesional isolate^d^ (TSST‐1 ug/ml, blister fluid^e^)	BP180 IgG ELISA	Diagnostic DIF	Severity, BPDAI	Patient ID	Assays^2^	Sex	Age, years	Skin isolate
E820^a^	MTS	M	72.6	TSST‐1+ (0.1630 ug/ml)	86	Linear IgG (1+) and C3 (3+)	43	E840	MTS	F	80.5	Coag neg
E811^a^	MTS	F	75.3	TSST‐1+	12	Linear IgG (1–2+) and C3 (1–2+)	11	E838	MTS	F	72	Coag neg
E810^a^	MTS	M	86.3	TSST‐1+ (0.0530 ug/ml)	164	Linear IgG (3+) and C3 (3+)	81	E830	MTS	M	71.6	No staph
E800^a^	MTS	M	78.2	Coag neg	99	Linear C3 (3+)	35	E854	MTS	F	75.5	Coag neg
E802^a^	MTS	M	74.6	TSST‐1+	21	ND	121	E852	MTS	M	87.2	Coag neg
E731^a^	MTS	F	81.6	TSST‐1+ (0.0075 ug/ml)	58	Linear IgG and C3	26	E839	MTS	M	77.7	S.aureus
E751^a^	MTS	F	72	Coag neg (4.1900 ug/ml)	85	Negative	80	E846	MTS, qPCR	M	76	Coag neg
E758^a^	MTS	M	67.2	*S. aureus* (0.0678 ug/ml)	179	Linear IgG and C3	95	E821	MTS, qPCR	M	81.5	Coag neg
E807^a^	MTS, qPCR	M	74.3	TSST‐1+ (0.0660 ug/ml)	44	Negative	95	E822	MTS, qPCR	M	66.1	No staph
E808^a^	MTS, qPCR	M	78.8	TSST‐1+	2	Linear IgG (2+) and C3 (3+)	173	E832	qPCR	M	69.1	No staph
E864	qPCR	M	85.6	TSST‐1+	2	ND	34	E825	qPCR	M	78.4	No staph
E866^a^	qPCR	F	86.5	TSST‐1+	6	Linear IgG (2+) and C3 (3+)	26	E833	qPCR	M	84.6	No staph
E896^a^	qPCR	F	64.5	TSST‐1+ (0.0920 ug/ml)	26	Linear IgG (1+) and C3 (+)	43	E835	qPCR	M	87.7	Coag neg
E901	qPCR	F	88.5	ND, no blister	21	ND	0	E823	qPCR	F	76.6	No staph
E904	qPCR	M	67.7	TSST‐1+	114	ND	30	E828	qPCR	F	85.6	No staph
E905	qPCR	F/Asian	45.4	ND, no blister	12	Linear IgG (1+) and C3 (3+)	0	E842	qPCR	M	81.1	Coag neg
E906	qPCR	F	91.3	ND, doxy	4	ND	5	E843	qPCR	M	90.3	Coag neg
E911	qPCR	F	93.4	TSST‐1+ (0.0183 ug/ml)	84	Negative	76	E844	qPCR	M	88.8	Coag neg
E869	FC	F	75.4	TSST‐1+	41	ND	38	E856	qPCR	F	75	No staph
E939	FC	F	74.4	ND, doxy	88	ND	ND	E859	qPCR	F	67.2	Coag neg
E617	FC	M	64.4	TSST1+ (1.0400 ug/ml)	69	Linear IgG and C3	59	E806	FC	F	48.8	No staph
E882	FC	M	73.2	*S. aureus*	75	Linear C3 (1+)	76	E858	FC	M	56	ND
E867	FC	M	80.4	TSST‐1+	3	ND	34	E950	FC	F	79.3	Coag neg
E798	FC	M	81.2	TSST‐1+	128	Linear IgG and C3	98	E847	FC	M	84.6	ND
S102	BrdU	F	88.6	TSST‐1+ (0.0091 ug/ml)	126	Linear IgG, C3, and fibrinogen	ND	S129	FC	F	69	*S. aureus*
E959	BrdU	M	77.5	ND, doxy	115	Linear IgG and C3	ND	E137	FC	M	71.7	ND
S101	BrdU	F	83.6	TSST‐1+	141	Linear IgG (2+) and C3 (2+)	63	S126	Flow, BrdU	F	58	Coag neg
E934	BrdU	M	82.3	ND	199	Linear IgG and C3	ND	E612	BrdU	M	72.8	Coag neg
E879	BrdU	F	63.7	Coag neg	51	ND	28	S122	BrdU	M	68.8	No staph
—	—	—	—	—	—	—	—	S124	BrdU	M	78.1	TSST‐1+ *S. aureus*
—	—	—	—	—	—	—	—	E818	BrdU	M	86.5	Coag neg
—	—	—	—	—	—	—	—	F144	BrdU	F	58	Coag neg
Summary:		52% M	Mean: 78	23/29 collected: 83% TSST‐1+ 6.9% *S. aureus* 13% Coag neg	Mean: 69	20/29 assayed: 87% positive 10.3% negative	Mean: 43	Summary:		63% M	Mean: 76	29/32 collected: 3.4% TSST‐1+ 6.9% *S. aureus* 55% coag neg 35% no staph

Abbreviations: BPDAI, Bullous pemphigoid Disease Area Index; coag neg, coagulase‐negative staphylococci; F, female; M, male; ND, not done; *S. aureus* , *Staphylococcus aureus* ; TSST‐1, toxic shock syndrome toxin‐1.

^a^ Colonisation was previously characterised [17].

^b^ MTS, proliferation via metabolic activity; BrdU, proliferation via DNA synthesis; qPCR, quantitative polymerase chain reaction; FC, flow cytometry.

^c^ Self‐reported race was Caucasian, unless indicated.

^d^ Staphylococcal isolates grown on blood agar plates were identified as catalase‐positive and coagulase‐positive *S. aureus* or catalase‐positive, coagulase‐negative staphylococci.

^e^ TSST‐1 quantitation was determined by immunoblot [17].

### 2.2 Bacterial Sampling and Staphylococcal Identification

Bacterial sampling and staphylococcal identification were conducted as previously described [17]. Briefly, swabs were streaked onto blood agar plates and incubated for 24 h (37°C, 5% CO^2^), and *S. aureus was* distinguished based on Gram‐positive staining and catalase‐negative and coagulase‐positive reactions.

### 2.3 Preparation of Peripheral Blood Mononuclear Cells

Peripheral blood mononuclear cells (PBMCs) were isolated using density gradient centrifugation. The PBMC layer was washed twice and resuspended in RPMI‐1640 supplemented with 5% dimethylsulfoxide (DMSO, VWR International, Radnor, PA), 20% normal human serum (Atlanta Biologicals, Flowery Branch, GA) by volume before freezing slowly overnight at −80°C and long‐term storage in liquid nitrogen.

For experiments, PBMCs were thawed quickly in pre‐warmed RPMI 1640 medium (Gibco, Thermo Fisher Scientific, Waltham, MA) containing 50 U/mL DNase (Benzonase nuclease; Sigma Chemical, St. Louis, MO), then washed and resuspended in RPMI 1640 containing 10% human serum (Gibco). Viable cells (3 × 10^6^/mL based on trypan blue exclusion) were rested for 24 h at 37°C and 5% CO_2_ without stimulation. Cells were then collected and counted, and samples with a viability > 90% were used in experiments.

### 2.4 Lymphocyte Proliferation Assays

Lymphocyte proliferation was evaluated using a standard in vitro stimulation assay [19]. Briefly, PBMCs were resuspended and plated at 1 × 10^5^ cells/well in a 96‐well plate. Cells were stimulated in triplicate with media alone (unstimulated), phytohemagglutinin (PHA, 2.5 μg/mL, ThermoFisher), or staphylococcal SAgs, TSST‐1, staphylococcal enterotoxin G (SEG), and staphylococcal enterotoxin‐like I (SEl‐I) (all at 0.1 μg/mL), produced and purified as previously described [20]. For MTS assays, cultures were incubated for 72 h, with reagent added during the final 4 h, and absorbance measured at 490 nm (Cell Titre 96 AQ_ueous_, Promega, Madison, WI). Alternatively, plates were incubated for 120 h, with BrdU added for the last 24 h (BrdU Cell Proliferation ELISA Kit, Abcam, Cambridge, MA), and the absorbance measured at 450 nm. Within each experiment, the triplicate mean absorbance values from the unstimulated wells were subtracted from the stimulated conditions.

### 2.5 CD4 ^+^ T Cell Enrichment, RNA Isolation, and cDNA Synthesis

Non‐adherent PBMCs were washed in PBS with 0.5% BSA and 2 mM EDTA (both Research Products International, USA), filtered through a 70 μm mesh, and CD4^+^ cells were enriched by immunomagnetic depletion (CD4^+^ T Cell Isolation Kit (human) Cat# 130–096‐533, Miltenyi Biotec, Auburn, CA). Cell purity of CD4^+^ cells (~87%) was confirmed via flow cytometry.

RNA was isolated using the RNeasy Mini Kit (Qiagen, Germany), followed by a Clean^+^ Concentrator kit (Zymo Research, Irvine, CA), and further purified and concentrated using a Zymo spin column with DNase digestion (Qiagen). RNA purity and quantity were evaluated with a NanoDrop (ThermoFisher). cDNA was synthesised using the SuperScript IV First Strand synthesis system (Invitrogen, Thermo Scientific) and stored at −20°C.

### 2.6 RT‐qPCR Primers, Experimental Design, and Analysis

Relative expression of Individual Vβ TCR chains was quantified by RT‐qPCR using published primer sets (Table S1) (Integrated DNA Technologies, Coralville, IA). Primer efficiency and reaction conditions were validated using serial dilutions of pooled healthy donor cDNA (Figure S1) [21].

Each experiment included three biological and three technical replicates. The baseline and threshold cycle (Ct) were determined using Design and Analysis software (Applied Biosystems, Thermo Fisher Scientific). Triplicate Ct values were averaged, normalised to GAPDH, and analysed using the 2‐ΔΔCt method [22]. No template and primer‐only controls were included in each run.

### 2.7 Flow Cytometry

Flow cytometry was performed using FACS buffer (PBS with 1% BSA, 0.1% sodium azide), with incubations in the dark at 4°C, unless specified otherwise. Antibody titrations and compensation were performed using UltraComp compensation beads (Cat# U20250, Invitrogen). The optimal concentrations of antibodies and viability stains were empirically determined (typically 2.5 to 5 μL/test).

To assess purity of CD4^+^ enriched cells, 1 × 10^6^ cells were stained with Ghost Violet 540 viability dye (Tonbo Biosciences Cat#13–0879‐T100, San Diego, CA), Fc block (FcX, BioLegend Cat# 422302, RRID:AB_2818986) and CD3‐Alexa Fluor700 (BioLegend Cat# 317340, RRID:AB_2563408), CD4‐Alexa Fluor488 (BioLegend Cat# 317420, RRID:AB_571939), and CD8‐PE (BioLegend Cat# 344724, RRID:AB_2562790), fixed, resuspended in FACS buffer and analysed using a BD LSR/Violet and FlowJo software (BD Biosciences v10.10.1, San Jose, CA).

For Vβ TCR analysis, PBMCs were stained with antibodies to CD3‐Alexa Fluor700 (BioLegend Cat# 317340, RRID:AB_2563408), CD8a‐PE‐Cy7 (Thermo Fisher Scientific Cat# 25–0088‐42), CD4‐BUV805 (Thermo Fisher Scientific Cat# 368–0047‐42), and 24 Individual Vβ‐FITC chains (Beta MarkTCR Vβ Repertoire Kit Beckman Coulter Cat# IM3497, RRID:AB_3696559), followed by fixation/permeabilisation (FOXP3/Transcription) Factor Staining Buffer Set (eBioscience Cat# 00–5523‐00) and then FoxP3‐PE‐CF594 (BD Biosciences Cat# 562421) staining. Data were acquired on a Cytek Aurora flow spectrometer (Cytek Biosciences, Fremont, CA) and analysed using FlowJo software (BD Biosciences v10.10.1, Boston, MA) to determine the percent Vβ expression on viable CD3^+^CD4^+^CD8^−^FoxP3^−^ (CD4^+^ T cells), CD3^+^CD4^+^CD8^−^FoxP3^+^ (Treg cells), and CD3^+^CD4^−^CD8^+^FoxP3^−^ (CD8^+^ T cells).

### 2.8 Statistical Analysis

Graphs were generated using GraphPad Prism version 10.10.1. Statistical tests included Fisher's exact test, unpaired *t*‐tests (or Mann–Whitney Test), and two‐way ANOVA as indicated. Data are expressed as mean ± standard error (SEM) or median ±95% confidence interval (CI). A *p*‐value of ≤ 0.05 was considered significant. If necessary, adjusted *p*‐values are shown and indicated in the figure legend.

## 3 Results

### 3.1 Patient Population

This study included 29 patients with BP (15 male/14 female, mean age [±SD] = 76.8 ± 10.1 years, range = 45.4–93.4 years) and 32 healthy controls (21 male/11 female, mean age [±SD] = 75.6 ± 9.7 years, range = 48.8–90.3 years). There were no significant differences in age (*p* = 0.6488, Mann–Whitney test) or sex (*p* = 0.3067, Fisher's exact test). Race was self‐reported: 28/29 BP patients identified as Caucasian, and 1 as Asian, while all 32 controls identified as Caucasian. Demographic, clinical, laboratory, and assay data for each participant are summarised in Table 1. Colonisation data for a subset of these patients were previously reported [17] and are identified in the table.

Bacterial swabs were available for 23/29 BP patients; three were excluded due to prior doxycycline treatment discovered after enrollment, and three were not sampled because there were no intact blisters. Of the analysed BP samples, 20 (87%) grew *S. aureus*, of which 19 (83%) were TSST‐1 positive. Among controls (29 sampled), staphylococcal growth was predominantly coagulase‐negative staphylococci (48%) coagulase‐negative (normal flora), with *S. aureus* identified in only two individuals (6.9%); the remaining samples (41%) showed no staphylococcal growth. Only one control (3.4%) carried TSST‐1^+^ *S. aureus* . These findings are consistent with prior observations of increased TSST‐1‐producing *S. aureus* colonisation in BP patients [17].

### 3.2 CD4 ^+^T Cell Proliferative Response to TSST‐1

*S. aureus* colonisation or infection with *S. aureus* can lead to SAg‐driven activation and expansion of peripheral T cells, which can be recapitulated in vitro [23, 24]. Given the high prevalence of TSST‐1‐producing *S. aureus* in BP patients, proliferative responses of PBMCs from BP patients and controls (10 each) were assessed following stimulation with TSST‐1 (0.10 μg/mL) or PHA (2.5 μg/mL).

**FIGURE 1:**
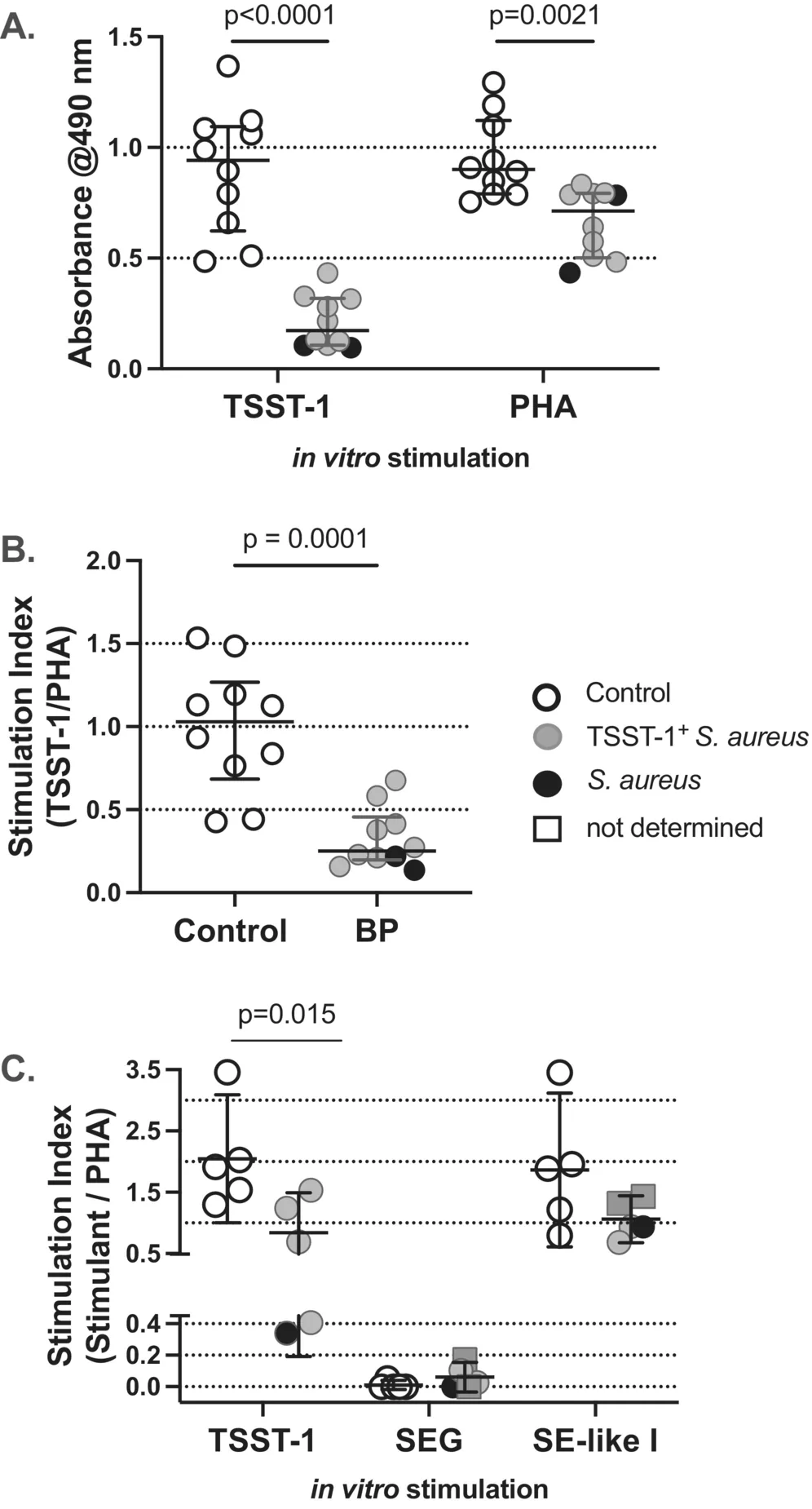
In vitro proliferative responses of PBMCs from BP patients and controls. PBMCs from BP patients and healthy controls were cultured in triplicate and stimulated with media alone, PHA (2.5 μg/mL), or *S. aureus* superantigens (TSST‐1, SEG, SEl‐I; 0.1 μg/mL each). Cells were incubated at 37°C with 5% CO_2_ for 72 h (A, B; MTS assay) or 120 h (C; BrdU assay). Triplicate values were averaged, and background (unstimulated) OD was subtracted. (A) PBMCs from controls showed greater proliferation than BP samples in response to PHA and TSST‐1. (B) Stimulation index analysis demonstrated reduced TSST‐1‐specific responses in BP PBMCs. (C) In an independent experiment, stimulation indices remained reduced in BP samples for TSST‐1 (adjusted *p* = 0.015), but not for SEG (*p* = 0.285) or SEl‐I (*p* = 0.222) (multiple Mann–Whitney tests). Each symbol represents an individual patient. Group differences were analysed using the Mann–Whitney test; adjusted *p*‐values are shown.

PBMCs from control subjects exhibited a robust proliferative response to both TSST‐1 and PHA, whereas PBMCs from BP patients demonstrated reduced proliferation (Figure 1A). Calculation of the stimulation index (TSST‐1/PHA) revealed significantly diminished TSST‐1 responsiveness of PBMCs from BP patients. (Figure 1B). Notably, the two patients without confirmed TSST‐1^+^ *S. aureus* colonisation (Figure 1, **black filled circles**) exhibited responses comparable to those with documented colonisation (Figure 1, **grey filled circles**).

Proliferative responses to additional *S. aureus* SAgs, SEG, and SEl‐I were then evaluated and compared with TSST‐1 (0.1 μg/mL each) (Figure 1C). In these experiments, proliferation was quantified by DNA synthesis (BrdU incorporation), rather than metabolic activity (MTS), to confirm assay robustness. While PBMCs from BP patients again exhibited reduced TSST‐1 responsiveness, no significant differences were observed between BP and control samples in response to SEG or SEl‐I. Together, these results indicate a selective reduction in TSST‐1‐induced proliferative responses in PBMCs from BP patients.

### 3.3 TCR Vβ Repertoire in Peripheral CD4 ^+^ T Cells

#### 3.3.1 TCR Vβ Gene Expression

Similarities in sequence or tertiary structures enable interaction of staphylococcal SAgs with one or more TCR chains, resulting in selective expansion of TCR Vβ subsets dictated by the expressed SAgs of the colonising strain [11, 24, 25, 26, 27]. For example, TSST‐1 drives the selective expansion of only TCR Vβ2^+^ lymphocytes [16], while other SAgs, such as SEG or SEl‐I, simultaneously stimulate several TCR Vβ subsets [25, 27]. To investigate whether BP patients exhibit alterations in the peripheral TCR Vβ repertoire, the relative expression of 23 individual TCR Vβ genes was measured by RT‐qPCR. cDNA was synthesised from CD4^+^ T cells enriched from PBMCs obtained from 10 BP patients and 14 healthy controls.

**FIGURE 2:**
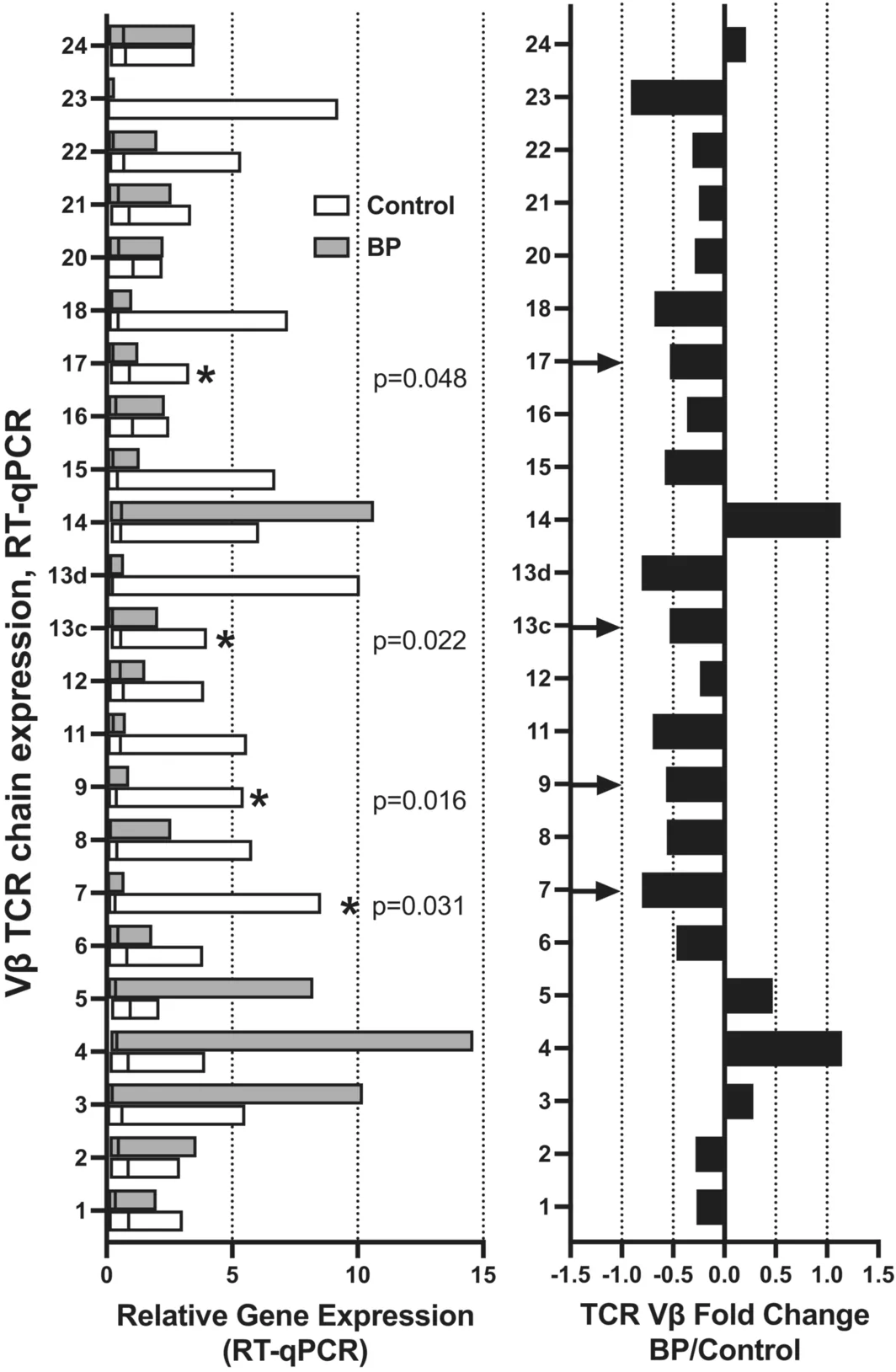
Peripheral TCR Vβ repertoire in *S. aureus* ‐colonised BP patients and controls. TCR Vβ and Cβ (control) gene expression were quantified in CD4^+^‐enriched peripheral T cells by RT‐qPCR. (A) Relative expression (normalised to GAPDH) is shown as floating bars (min–max) with the median. Vβ 7, 9, 13c, and 17 were significantly reduced in BP samples (Mann–Whitney test with adjusted *p*‐values). (B) Fold change in Vβ expression relative to controls, normalised to Cβ. Arrows show that the significant differences in panel (A) do not meet the ≥ 2‐fold change threshold to be biologically meaningful.

Using this approach, no significant increases in TCR Vβ expression were observed in bulk CD4^+^ T cells from BP patients compared with controls. Specifically, TCR Vβ2 was not increased in BP patients, despite the high prevalence of TSST‐1^+^ *S. aureus* colonisation (Figure 2A). In contrast, relative expression of Vβ7, 9, 13c, and 17 was significantly higher in control CD4^+^ T cells (*p* = 0.016 to 0.048). Despite these differences, fold change analysis [22] did not identify any biologically meaningful (two‐fold) differences in Vβ expression between the two groups (Figure 2B).

Because gene expression differences can be obscured by mixed cell populations and do not necessarily reflect protein‐level expression, the functional TCR Vβ repertoire was subsequently evaluated by surface immunostaining and flow cytometry.

#### 3.3.2 TCR Vβ Cell Surface Expression on T Cell Subsets

Multicolour immunostaining of PBMCs permitted evaluation of 24 individual TCR Vβ chains on the surface of conventional CD4^+^ T cells (CD3^+^CD4^+^CD8^−^FoxP3^−^), Treg cells (CD3^+^CD4^+^CD8^−^FoxP3^+^), and CD8^+^ T cells (CD3^+^CD4^−^CD8^+^FoxP3^−^) using PBMCs from 6 BP patients and 6 controls. All three of these T cell subsets are susceptible to SAg‐mediated expansion and modulation of the TCR Vβ repertoire.

**FIGURE 3:**
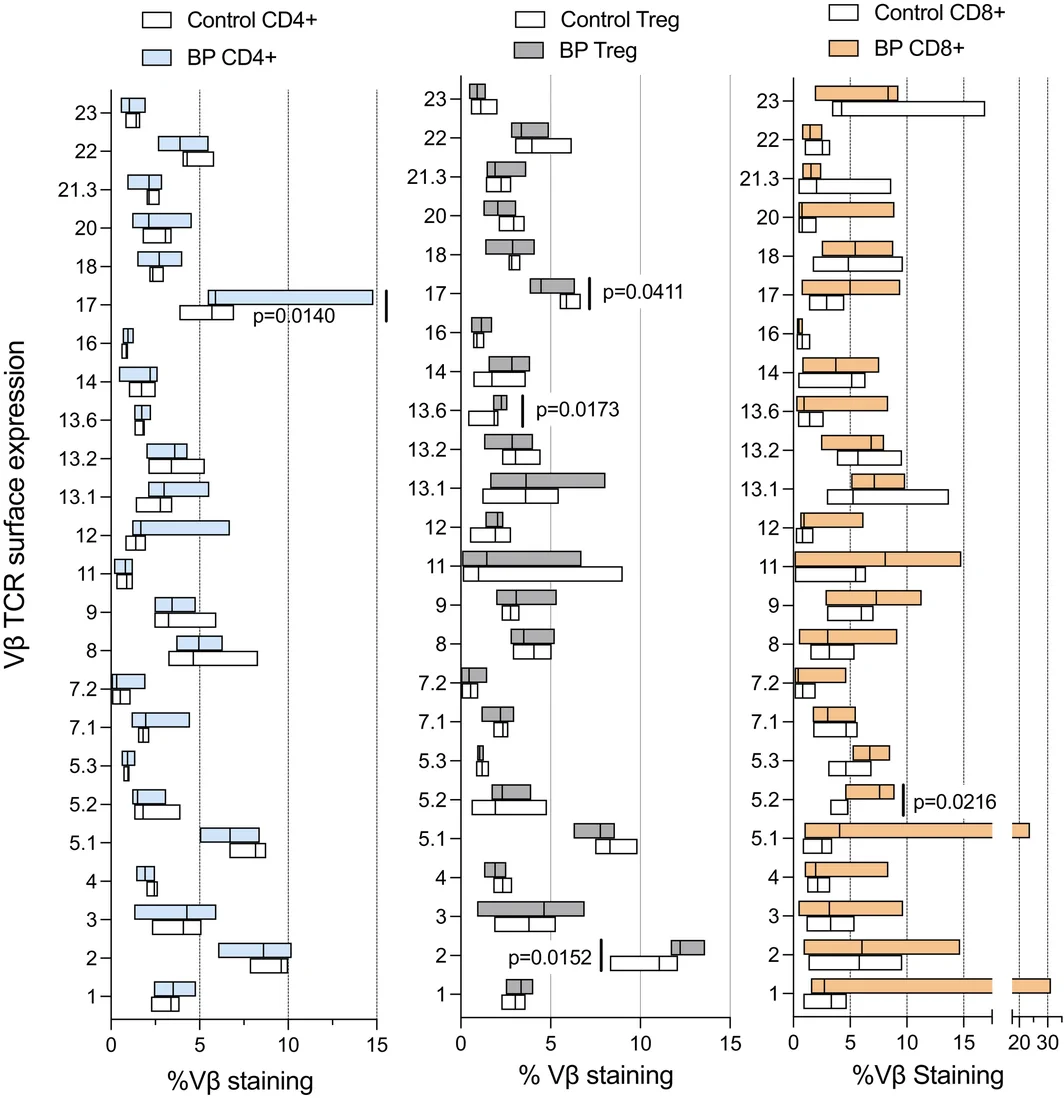
Peripheral TCR Vβ repertoire utilisation in *S. aureus* colonised BP patients vs. healthy controls. TCR Vβ usage was assessed by flow cytometry following immunostaining of PBMCs from 6 BP patients and 6 healthy controls. Per cent positivity for each Vβ is shown as floating bars (min–max, median line). (A) conventional CD4^+^FoxP3^−^ T cells, (B) Regulatory T cells (CD4^+^FoxP3^+^), (C) CD8^+^CD4^−^FoxP3^−^ T cells. Group differences were evaluated using the unpaired Mann–Whitney test; exact *p*‐values are shown where significant.

Since *S. aureus* can express any combination of > 20 SAg toxins, we anticipated expansion of multiple TCR Vβ subsets in BP patients. However, flow cytometric analysis revealed only limited, subset‐specific differences in Vβ usage (Figure 3). Surprisingly, but consistent with our qPCR results, the percentage of TCR Vβ2^+^ CD4^+^ T cells was not increased in BP patients compared to controls. In fact, the only specific Vβ expansion noted in CD4^+^ T cells from BP patients was an increase in Vβ17 usage (*p* = 0.0140), although its high inter‐individual variability limits its overall impact (Figure 3A).

On the other hand, Vβ2 expression was significantly (*p* = 0.0152) increased in Tregs from BP patients compared to controls, as was Vβ 13.6 (*p* = 0.0173), while the percentage of Vβ17+ Tregs was higher in controls (*p* = 0.0411) (Figure 3B). Finally, CD8^+^ T cells from BP patients were characterised by a high degree of inter‐individual variability in Vβ repertoire utilisation (Figure 3C), with a significant increase observed only in the Vβ5.2 subset (*p* = 0.0216). The Vβ2 populations were similar in CD8^+^ T cells from BP patients and controls.

Since the differences in repertoire in BP patients vs. controls were not consistent across T cell subsets, we next compared Vβ usage among conventional CD4^+^, Treg, and CD8^+^ T cell subsets from BP patients (Table 2). Of 24 Individual Vβ chains, only 12 (2, 5.1, 5.2, 5.3, 7.2, 9, 13.1, 13.2, 13.6, 16, 22, and 23) differed significantly between the T cell subsets. The most notable finding was that the Vβ2 population was significantly increased in Tregs, compared with either CD4^+^ or CD8^+^ T cells. In fact, Vβ2 was the only Vβ subset for which expression was highest in Tregs.

**TABLE 2:** Significant comparisons of TCR Vβ chain expression on peripheral T cell subsets from BP patients ^a^ ^,^ ^b^.

TCR	ANOVA	CD4+ vs. Treg	CD4+ vs. CD8+	Treg vs. CD8+
Vβ2	0.0022	0.0447	ns	0.0446
Vβ5.1	0.0109	0.0148	ns	ns
Vβ5.2	< 0.0001	ns	0.0007	ns
Vβ5.3	0.0003	ns	0.0042	0.0240
Vβ7.2	0.0043	0.0088	ns	ns
Vβ9	0.0231	ns	ns	0.0449
Vβ13.1	0.0139	ns	0.0242	ns
Vβ13.2	0.0249	ns	ns	0.0331
Vβ13.6	0.0186	ns	ns	0.0206
Vβ16	0.0078	ns	ns	0.0240
Vβ22	0.0003	ns	0.0043	0.0242
Vβ23	0.0011	ns	0.0175	0.0105

^a^ VβTCR chains were measured on T cell subsets using immunofluorescent staining and flow cytometry. T cell populations were CD4^+^ T cells (CD3^+^CD4^+^CD8^−^FoxP3^−^), Treg cells (CD3^+^CD4^+^CD8^−^FoxP3^+^), and CD8^+^ T cells (CD3^+^CD4^−^CD8^+^FoxP3^−^).

^b^ Kruskal–Wallis non‐parametric ANOVA with Dunn's correction for multiple comparisons; exact *p*‐values shown. ns, not significant.

## 4 Discussion

Studies from our laboratory and others have demonstrated a high rate of *S. aureus* colonisation in BP lesions that correlates with disease activity [17, 18]. In some clinical contexts, antibiotic treatment reduces this activity [17], suggesting that staphylococcal colonisation actively drives pathogenesis rather than merely reflecting barrier disruption. The objective of this study was to determine whether *S. aureus* colonisation in BP patients is associated with SAg‐related alterations in peripheral T cell responses, with particular attention to proliferative capacity and TCR Vβ repertoire composition.

We hypothesised that BP patients might exhibit SAg‐mediated expansion of peripheral CD4^+^ T cells, detectable through enhanced proliferation in response to TSST‐1 or enrichment of TCR Vβ2^+^ T cells. Contrary to this hypothesis, PBMCs from BP patients exhibited attenuated proliferative responses to TSST‐1 and no marked expansion of Vβ2+ conventional CD4^+^ T cells. These findings indicate that systemic T cell responsiveness to TSST‐1 is reduced rather than enhanced in BP.

Several non‐mutually exclusive mechanisms may account for this reduced responsiveness. One possibility is that widespread exposure to TSST‐1^+^ *S. aureus* earlier in life may lead to the development of neutralising antibodies, limiting effective systemic exposure in older BP patients [11, 28]. Consistent with this notion, BP patients frequently harbour TSST‐1^+^ *S. aureus* in lesions and detectable toxin in blister fluid, without overt clinical signs of infection [17].

Alternatively, chronic colonisation and prolonged SAg exposure may promote functional hypo‐responsiveness, including T cell anergy, deletion of responsive clones, or the activation of regulatory pathways that suppress SAg‐specific responses [12, 13]. Such mechanisms could result in reduced host responsiveness to *S. aureus* and impaired antimicrobial responses, an important clinical concern given that infectious complications account for substantial morbidity and mortality in BP [29].

In support of this regulatory bias, we observed selective enrichment of Vβ2^+^ Tregs without a corresponding expansion in conventional CD4^+^ or CD8^+^ T cell subsets. Circulating Tregs are known to respond to TSST‐1 (and other SAgs), while maintaining FoxP3 expression and suppressive activity, including inhibition of TSST‐1‐induced proliferation of conventional T cells [30, 31]. However, the regulation of these expanded Vβ2^+^ Tregs was not tested directly in this study because functional suppression assays and cytokine profiling were not feasible. Consequently, conclusions regarding immune tolerance remain inferential, and this enrichment should be interpreted as phenotypic rather than functional evidence of regulation.

Importantly, these observations are derived from peripheral blood, and the immune process within lesional skin may differ substantially. Cytokines and other soluble mediators are enriched in the skin and blister fluid, where they interact directly with keratinocytes and tissue‐resident immune cells, generating a highly localised inflammatory microenvironment [32]. While peripheral blood is commonly used as a surrogate in skin disease research and may reflect systemic components of cutaneous inflammation, mechanistic conclusions regarding skin‐localised pathogenesis drawn from circulating cells remain indirect. Tissue‐restricted TCR Vβ skewing has been reported in several immune‐mediated diseases, including enrichment of specific Vβ subsets in synovial fluid in rheumatoid arthritis [33, 34, 35] and in lesional skin in autoimmune and inflammatory dermatoses [36, 37]. Tissue‐specific shaping of the TCR repertoire by local microbial communities is also well documented [38]. These observations underscore the need for direct analysis of skin‐resident immune populations.

Several limitations warrant consideration. First, BP is a rare disease primarily affecting elderly individuals with multiple comorbidities, resulting in unavoidable clinical heterogeneity with respect to disease activity, medications, and microbial exposure. Because our institution is a tertiary referral centre, many patients were evaluated after initiation of therapy, limiting control over treatment status. This treatment‐related and inter‐individual variability may have contributed to the observed range of immune responses. In addition, variable and generally low cell yields from elderly patients constrained sample availability, thereby reducing statistical power. Further, the high prevalence of TSST‐1^+^ *S. aureus* colonisation limited our ability to compare colonised vs. uncolonised BP patients; therefore, more investigation is needed to provide definitive conclusions.

Finally, the single‐centre design and Midwestern referral population resulted in a predominantly non‐Hispanic Caucasian cohort. While SAg expression by *S. aureus* varies with genetic and environmental factors [15], the consistently high prevalence of TSST‐1‐producing strains observed across multiple BP cohorts suggests that this observation may not be restricted to a single geographic or demographic population. Nevertheless, broader multi‐centre studies will be required to assess generalisability.

In summary, our study demonstrates that peripheral T cells in BP patients do not exhibit Vβ2^+^ expansion or heightened responsiveness typically associated with TSST‐1, despite frequent colonisation with TSST‐1‐producing strains. Instead, TSST‐1‐induced proliferation is attenuated, and Vβ2^+^ T cells are selectively enriched within the Treg compartment. Together, these findings are consistent with enhanced regulatory features limiting peripheral SAg reactivity, while leaving open the possibility that distinct immune mechanisms operate within lesional skin. Future studies integrating direct skin analysis and functional assays will be required to delineate the role of SAg‐exposed immune cells in BP pathogenesis.

## Author Contributions

Conceptualisation and Design: K.N.M., J.A.F. Investigation: T.Y.C., T.P.C., M.F., M.P., S.H.K. Visualisation and Formal Analysis: T.Y.C., T.P.C., K.N.M. Resources, Supervision: K.N.M., P.M.S., J.A.F. writing, original draft: T.Y.C., T.P.C. writing, critical revision: T.Y.C., K.N.M. writing, review, and editing: T.Y.C., T.P.C., M.F., M.P., K.N.M., J.A.F. All authors have read and approved the final manuscript.

## Funding

This work was supported by the National Institutes of Health (R21AG065980, UL1TR002537, and T32AI007343), the University of Iowa, the Roy J. and Lucille A. Carver College of Medicine, the University of Iowa, and the University of Iowa Department of Dermatology. The funding agencies did not influence the design, interpretation, writing, or submission of this study for publication. The content is solely the responsibility of the authors and does not necessarily represent the official views of the funding agencies.

## Ethics Statement

Reviewed and approved by the University of Iowa Institutional Review Board, approval #2016077048, #202008473.

## Conflicts of Interest

The authors declare no conflicts of interest.

## Supporting information


**Table S1:** qPCR primer sequences (5′ to 3′) for TCR Vβ analysis.
**Figure S1:** CD4+ T enrichment and validation of Vβ TCR primers and qPCR reaction conditions. CD4+ T cells were enriched from PBMCs by immunomagnetic negative selection. (A) Pre‐ and post‐separation cells were immunostained and analysed by flow cytometry. Cells were gated on FSC vs. SSC, Singlets, Live/Dead, and CD3+. An average of 87% live CD4+/CD3+ cells were obtained. (B) Serial dilutions of a standard cDNA sample, derived from pooled PBMCs from healthy volunteers, were used to validate conditions for constant β (Cβ) and 23 Individual Vβ TCR chains. The averaged Ct values from three technical replicates of the Log of cDNA (ng/reaction) for primer pairs corresponding to each TCR Vβ chain were used to generate slopes and correlation coefficients (R2). The midpoint of the linear dynamic range indicated that seven ng total cDNA/reaction was optimal for all primer sets.

## Data Availability

Data sharing is not applicable to this article as no datasets were generated or analysed during the current study.
